# Sexuality experiences of secondary school students in Nakuru, Kenya: a cross-sectional study

**DOI:** 10.4314/ahs.v18i2.3

**Published:** 2018-06

**Authors:** Tammary Esho, Arun Datta, Samuel Muniu

**Affiliations:** 1 Technical University of Kenya, Community and Public Health; 2 Technical University of Kenya, Centre for Science and Technology Studies

**Keywords:** Sexuality experiences, secondary school students, Nakuru, Kenya

## Abstract

**Background:**

Adolescence is a phase where one is inquisitive about sex and sexuality. It is but natural to exchange the half-baked feelings and experiences with peers. These days the environment that includes public media has sexually suggestive flavors.

**Objective:**

This study was conducted to find out the sexual experiences of a selected, few school going adolescents.

**Methods:**

A cross-sectional study utilizing stratified sampling to enroll 200 students from secondary schools in Nakuru County, Kenya. A self-administered questionnaire was used to collect data. The data was entered and analyzed in SPSS® version 22. Chi-square test of independence and Fisher's Exact Test were performed to test for associations.

**Results:**

The study found that a large number have had sexual experiences of varying type. It was interesting to learn from the survey that a large number of older adolescents wish that their parents should have talked to them about sexual matters. There is a general conservative outlook that the students had. Although a small percentage had sexual experiences at a very young age, many of them valued ethics and morality.

**Conclusion:**

There is urgency for intervention by the parents and the church in filing the gap in sexual knowledge.

## Introduction

Globally, reproductive health of adolescents is a matter of great concern. Adolescence is a transition stage where major developments take place and one of the most complex processes is sexual maturation and onset of over sexual behaviour. At this stage the adolescents gain autonomy from their parents, develop a network of peers and begin to pursue romantic connections^1^. Early adolescence (10 – 13 years) is characterized by different behavioral experimentation, middle adolescence (14 – 16 years) is full of risk taking, while commencement of risk-taking occurs in late adolescence (17 – 19 years)^2^.

The bio-psycho-social model explains adolescent sexual development^3^. The biological factors are comprised of the neuro-endocrinal and genetic factors. Several physiological changes occur such as development of pubic hair and commencement of menstruation in females. All these processes are driven by hormones which also significantly influences the development of sexual interests among the adolescents^2^.

Another important aspect relates to psychological factors where personal characteristics or temperament changes in a way that shapes attitudes toward sexuality^4^. Social or environmental factors also shape attitudes of adolescents on sexuality besides facilitating sexual learning. Such social factors include peer relations, parental attitudes towards sexuality, culture and parenting style^5^,^6^. With modernization and urbanization, mass media also significantly influences sexuality in adolescents^7^. Lately the exposure of adolescents to material with sexual content in movies and over the internet has had an impact on their perception about sexuality.

The experimentation tendency of adolescents leads them to engage in risky behaviors such as having unprotected sex, multiple sexual partners, sexual debut at a very early age and having sex under the influence of drugs^8^. It is these risky behaviors initiated during adolescence that predispose adolescents to health hazards such as contracting sexually transmitted diseases and contribute to increased cases of unintended pregnancies^9^.

According to World Health Organization (WHO), adolescent pregnancies pose a great threat to life and lead to a higher proportion of maternal and child mortality^10^. In low and middle income countries 17 million girls under the age of 19 give birth of which 1 million are under 15 years. It is worth noting that globally, the second cause of mortality for girls aged 15 to 19 is pregnancy and childbirth related complications^10^. This may be attributed to unsafe abortions. The risk of still births or death is higher (50%) among babies born to adolescent mothers as compared to those born to mothers aged 20 – 29 in low and middle income countries^10^.

Kenya is home to 10.5 million adolescents aged 10–19 years representing 22.5% of the total population^11^. The mean age at which adolescents have their first baby in Kenya is 17.4 and 18.1 years for girls and boys, respectively. In addition, about 1.5 million Kenyan adolescents aged 15–19 are sexually active, with the mean age of having the first sexual intercourse as 16.3 and 15.5 years for girls and boys, respectively^11^. Considering that a good proportion of adolescents in Kenya are in secondary schools, it's important to elucidate their sexual experiences.

A majority of the secondary school students in Kenya range from age 14 to 18 years. The curriculum of the secondary school in Kenya does not explicitly cover sexuality or sexual health education. The subject is probably more or less briefly covered within other subjects such as biology. This study was conducted to investigate the sexual behavior and experiences of secondary school students who are in their formative years of life. Findings from this study will provide useful information to parents, school teachers, counselors and church leaders. It will help them to address the needs of the adolescents and youth in terms of supporting and instilling morality and ethics with regards to sexuality.

## Methods

### Study settings

The study was conducted in Nakuru, the fourth largest county in Kenya. It is a cosmopolitan county with almost all Kenyan ethnic groups broadly represented. According to the 2009 census, the county is home to 1.6 million people^12^. It has a considerable proportion of youthful population. The adolescent (10 – 19 years) makes up a fifth of the people residing in the county^13^. Such youthful population increases demand for education and health services thereby affecting the development agenda of the county.

### Study design

This was a cross-sectional study. The study aimed to investigate the sexual behavior and experiences of adolescents at the time of data collection. The design is appropriate in collecting data from a large number of respondents in a relatively short time. It is also relatively inexpensive.

### Sample size and sampling

Using stratified sampling procedure, 68 students were enrolled into the study from the 200 that had attended the mentorship program. Representation of gender and the schools that had attended the program were the factors considered in the sampling procedure.

### Study subjects

The study respondents were secondary school students who had attended a mentorship program organized at a secondary school in Nakuru County, Kenya. The students were peer leaders from 10 secondary schools. The aim of the mentorship program was to build capacity of the peer leaders so that they can support other students in their schools.

### Study instrument

A questionnaire with open-ended questions was used to collect data. The questionnaire captured personal information such as age, gender and religion. It also captured information on sexuality. The questions on sexuality included; the first person they talk to about sex, people they wish to talk to about sex and sexuality matters, their source of information about sex, and whether they had engaged in sexual behavior.

### Data collection

Data collection took place at the venue of the mentorship program. The students were taken through informed consent before data collection. Students that consented to participate in the study were each given a questionnaire and requested to respond to the questions appropriately. The data was collected before the commencement of the mentorship program. The students took approximately 20 minutes to complete the questionnaire.

### Ethical considerations

For confidentiality purposes, the names of the respondents were not captured during data collection. The students were not coerced into answering any questions. Permission was sought to conduct the study from the respective school administrations.

### Data management and statistical analyses

The data was entered and analyzed in Statistical Package for Social Sciences (SPSS®) version 22. Numerical data was analyzed into mean and standard deviations while categorical into proportions and summarized in a table. Chi-square test of independence and Fisher's Exact Test were performed to test for associations. A p-value of 0.05 was considered statistically significant.

## Results

### Demographic characteristics in relation to respondents engagement in sexual behavior

The study participants comprised 68 secondary school students. The average age of the participants was 16.62 ± 1.44 (Mean ± SD) years. With regard to secondary class level, the participants were distributed as follows: form one 16 (23.5%), form two 20 (29.4%), form three 19 (27.9%) and form four 13 (19.1%). In terms of gender, the participants were almost equally distributed (Table 1). The majority (91.2%, n = 62) of the participants were Christians. Overall, 44.1% (n = 30) had engaged in sexual behavior at the time of the study. Chi-square test of independence was performed and revealed no statistical correlation between age, gender and respondents engagement in sexual behavior.

**Table 1:** Demographic characteristics of the respondents in relation to sexual behavior

Have engaged in sexual behavior				
Variable	Yes (%)	No (%)	Total (%)	p-value
Age				0.103
14 – 16 years	13 (35.1)	24 (64.9)	37 (100.0)	
17+ years	17 (54.8)	14 (45.2)	31 (100.0)	
Gender				0.829
Male	15 (45.5)	18 (54.5)	33 (100.0)	
Female	15 (42.9)	20 (57.1)	35 (100.0)	
Total	30 (44.1)	38 (55.9)	68 (100.0)	

Type of sexual behavior engaged					
Variable	Kissing (%)	Touching body parts (%)	Intercourse (%)		
Age					0.621
14 – 16 years	7 (53.8)	3 (23.1)	3 (23.1)	13 (100.0)	
17+ years	9 (52.9)	6 (35.3)	2 (11.8)	17 (100.0)	
Gender					0.034
Male	5 (33.3)	5 (33.3)	5 (33.3)	15 (100.0)	
Female	11 (73.3)	4 (26.7)	0 (0.0)	15 (100.0)	
Total	16 (53.3)	9 (30.0)	5 (16.7)	30 (100.0)	

Age at first ever sexual encounter					
Variable	6 – 10 years (%)	11 – 15 years (%)	16 – 20 years (%)		
Age					0.07
14 – 16 years	1(7.7)	8(61.5)	4(30.8)	13(100)	
17+ years	1(5.9)	4(23.5)	12(70.6)	17(100)	
Gender					0.243
Male	2(13.3)	7(46.7)	6(40.0)	15(100)	
Female	0(0.0)	5(33.3)	10(66.7)	15(100)	
Total	2 (6.7)	12 (40.0)	16 (53.3)	30 (100)	

Analysis with Chi-Square test of independence

### Sexuality information

Nearly half (48.5%, n = 33) of the respondents indicated that the first person who talked to them about sex was one of their friends (Table 2).

**Table 2:** Communication about sex and opinion about marriage

First person who talked to me about sex							
Variable	Parents (%)	Friends (%)	Teachers (%)	No one (%)	Siblings (%)	Total (%)	p-value
Age							0.467
14 – 16 years	6 (16.2)	17 (45.9)	9 (24.3)	4 (10.8)	1 (2.7)	37 (100.0)	
17+ years	9 (29.0)	16 (51.6)	4 (12.9)	2 (6.5)	0 (0.0)	31 (100.0)	
Gender							0.041
Male	4 (12.1)	22 (66.7)	5 (15.2)	2 (6.1)	0 (0.0)	33 (100.0)	
Female	11 (31.4)	11 (31.4)	8 (22.9)	4 (11.4)	1 (2.9)	35 (100.0)	
Total	15 (22.1)	33 (48.5)	13 (19.1)	6 (8.8)	1 (1.5)	68 (100.0)	

Talk to about sex								
Variable	Parents (%)	Friends (%)	Teachers (%)	Pastor (%)	Doctors (%)	No one (%)		
Age								0.369
14 – 16 years	5 (13.5)	18 (48.6%)	4 (10.8)	1 (2.7)	0 (0.0)	9 (24.3)	37 (100.0)	
17+ years	3 (9.7)	20 (64.5)	2 (6.5)	0 (0.0)	2 (6.5)	4 (12.9)	31 (100.0)	
Gender								0.24
Male	1 (3.0)	21 (63.6)	3 (9.1)	1 (3.0)	1 (3.0)	6 (18.2)	33 (100.0)	
Female	7 (20.0)	17 (48.6)	3 (8.6)	0 (0.0)	1 (2.9)	7 (20.0)	35 (100.0)	
Total	8 (11.8)	38 (55.9)	6 (8.8)	1 (1.5)	2 (2.9)	13 (19.1)	68 (100.0)	

Opinion about marriage							
Variable	I don't mind (%)	I like (%)	I fear (%)	I hate (%)	Anger (%)		
Age							0.537
14 – 16 years	11(29.7)	10(27.0)	11(29.7)	4(10.8)	1(2.7)	37(100)	
17+ years	6(19.4)	13(41.9)	10(32.3)	1(3.2)	1(3.2)	31(100)	
Gender							0.04
Male	9(27.3)	16(48.5)	7(21.2)	1(3.0)	0(0.0)	33(100)	
Female	8(22.9)	7(20.0)	14(40.0)	4(11.4)	2(5.7)	35(100)	
Total	2(2.9)	1(1.5)	3(4.4)	54(79.4)	8(11.8)	68(100)	

Analysis with Chi-Square test of independence

Chi-square test of independence revealed that there is a significant difference (p = 0.041) between gender and the first person to talk to them about sex. The male participants were more likely to have their friends talking to them about sex. However more female participants had parents talk to them about sex.

With regard to the person the respondents wish to talk to about sex and sexuality matters, a higher number indicated parents (48.5%, n = 33). Although not statistically different, more participants aged 17 years and above and females wished to talk to parents about sex and sexuality matters (Table 3).

**Table 3:** The person the respondents wish to talk to about sex and sexuality matters, source of sex information and reasons for not engaging in sexual behaviour

Wish to talk to about sex and sexuality matters									
Variable	Parents (%)	Friends (%)	Teachers (%)	Pastor (%)	No one (%)	Future husband (%)	Motivational speaker (%)	Total (%)	p-value
Age									0.718
14 – 16 years	16 (43.2)	7 (18.9)	9 (24.3)	0 (0.0)	3 (8.1)	1 (2.7)	1 (2.7)	37 (100.0)	
17+ years	17 (54.8)	6 (19.4)	4 (12.9)	1 (3.2)	3 (9.7)	0 (0.0)	0 (0.0)	31 (100.0)	
Gender									0.163
Male	12 (36.4)	7 (21.2)	7 (21.2)	1 (3.0)	5 (15.2)	0 (0.0)	1 (3.0)	33 (100.0)	
Female	21 (60.0)	6 (17.1)	6 (17.1)	0 (0.0)	1 (2.9)	1 (2.9)	0 (0.0)	35 (100.0)	
Total	33 (48.5)	13 (19.1)	13 (19.1)	1 (1.5)	6 (8.8)	1 (1.5)	1 (1.5)	68 (100.0)	

Source of sex information									
Variable	Books (%)	Internet (%)	Magazines (%)	Friends (%)	Guidance and counseling (%)	Parents (%)	Television (%)		
Age									0.395
14 – 16 years	10 (27.0)	13 (35.1)	9 (24.3)	2 (5.4)	1 (2.7)	0 (0.0)	2 (5.4)	37 (100.0)	
17+ years	4 (12.9)	15 (48.4)	8 (25.8)	3 (9.7)	0 (0.0)	1 (3.2)	0 (0.0)	31 (100.0)	
Gender									0.005
Male	3 (9.1)	20 (60.6)	6 (18.2)	3 (9.1)	0 (0.0)	1 (3.0)	0 (0.0)	33 (100.0)	
Female	11 (31.4)	8 (22.9)	11 (31.4)	2 (5.7)	1 (2.9)	0 (0.0)	2 (5.7)	35 (100.0)	
Total	14 (20.6)	28 (41.2)	17 (25.0)	5 (7.4)	1 (1.5)	1 (1.5)	2 (2.9)	68 (100.0)	

Reasons for not engaging in sexual behavior									
Variable	It is unethical and immoral (%)	It can cause unwanted pregnancies (%)	It can cause incurable diseases (%)	I have no desire (%)	Hindrance to my goals (%)	Value virginity (%)	Waiting for right time (%)		
Age									0.87
14 – 16 years	9 (37.5)	3 (12.5)	4 (16.7)	5 (20.8)	1 (4.2)	1 (4.2)	1 (4.2)	24 (100.0)	
17+ years	8 (57.1)	2 (14.3)	3 (21.4)	1 (7.1)	0 (0.0)	0 (0.0)	0 (0.0)	14 (100.0)	
Gender									0.455
Male	9 (50.0)	3 (16.7)	3 (16.7)	1 (5.6)	0 (0.0)	1 (5.6)	1 (5.6)	18 (100.0)	
Female	8 (40.0)	2 (10.0)	4 (20.0)	5 (25.0)	1 (5.0)	0 (0.0)	0 (0.0)	20 (100.0)	
Total	17 (44.7)	5 (13.2)	7 (18.4)	6 (15.8)	1 (2.6)	1 (2.6)	1 (2.6)	38 (100.0)	

Analysis with Chi-Square test of independence

More than half (55.9%, n = 38) of the respondents talked to friends about sex (Table 2). Although not statistically significant, more respondents aged 17 years and above than their younger counterparts and more males than females talked to friends about sex. It is worth noting that only 11.8% (n = 8) of the respondents, actually talked to parents about sex. Female students were more likely to talk to parents about sex than males although the difference was not statistically significant.

Internet was indicated by a high number (41.2%, n = 38) of the respondents as the source of sex information (Table 3). Those aged 17 years and above were more likely to use internet as source of sex information than their younger counterparts although the difference was not statistically significant. Fisher's Exact Test revealed a significant relationship (p = 0.005) between gender and source of sex information. More male students were likely to use internet as the source of sex information.

Of the 44.1% (n = 30) respondents that had engaged in sexual behavior, more than half (53.3%, n = 16) had engaged in kissing (Table 1). It was noticed that gender is significant (p = 0.034) in so far as the type of sexual behavior is concerned. More females engaged in kissing. However, it was further noticed that it is the males who engaged in intercourse. No significant relationship was found between age and the type of sexual behavior engaged.

Ethical and moral grounds were cited for non-indulgence in sexual activity by a high proportion (44.7%, n = 17) of the respondents that had not engaged in sexual behavior (Table 3). Interestingly, virginity was of no importance among the respondents. Noticeably, a small percentage is concerned about unwanted pregnancies. The fear of incurable diseases is just slightly more of a deterrent. Achievement of goal and waiting for the right partner are insignificant in so far as sexual activities are concerned.

More than half (53.3%, n = 16) of respondents who had engaged in sexual behavior, had their first sexual encounter when they were between 16 – 20 years of age (Table 1). Male respondents tend to have their first sexual encounter at an earlier age than females, although the difference is not statistically significant.

The study elicited perceptions (feelings) about love, marriage, pornography, masturbation and homosexuality. In general, the majority (64.7%, n = 44) indicated that they like [romantic] love. A third (33.8%, n = 23) reacted to favorably marriage. A large (72.1%, n = 49) proportion of the respondents reported that they hate pornography (Table 4).

**Table 4:** Reactions to concepts of “love, marriage, pornography, masturbation and homosexuality”

Reaction to	I don't mind (%)	I like (%)	I fear (%)	I hate (%)	Anger (%)	Total (%)
Love	10 (14.7)	44 (64.7)	7 (10.3)	5 (7.4)	2 (2.9)	68 (100)
Marriage	17 (25)	23 (33.8)	21 (30.9)	5 (7.4)	2 (2.9)	68 (100)
Pornography	4 (5.9)	4 (5.9)	5 (7.4)	49 (72.1)	6 (8.8)	68 (100)
Masturbation	7 (10.3)	3 (4.4)	4 (5.9)	44 (64.7)	10 (14.7)	68 (100)
Homosexuality	2 (2.9)	1 (1.5)	3 (4.4)	54 (79.4)	8 (11.8)	68 (100)

Many (64.7%, n = 44) hated masturbation. Almost 15% seemed to be indulging in it and a similar percentage going to the other extreme of hating it. Again a large number (79.4%, n = 54) of the respondents hated homosexuality. Surprisingly 11.8% responded to homosexuality with anger and almost 3% did not mind it.

Reactions to marriage were found to be significantly (p = 0.04) related to gender but not to age. More males reacted positively to marriage than females (Table 2). On the contrary, more females reacted with fear to marriage than males. However, the reaction to concepts of love, pornography, masturbation and homosexuality in relation to age and gender were found not statistically significant. The respondents used the internet to access information easily on the concepts of; love (58.8%, n = 40), marriage (17.6%, n = 12), pornography (36.8%, n = 25), masturbation (7.4%, n = 5) and homosexuality (13.2%, n = 9) (Table 5). Chi-square test of independence revealed a significant (p < 0.001) relationship between gender and use of internet to search for information on pornography. More males reported using internet for pornography.

**Table 5:** Use of internet

Used internet to get information about concepts of;	Love (%)		Marriage (%)		Pornography (%)		Masturbation (%)		Homosexuality (%)		Total (%)

	Yes	No	Yes	No	Yes	No	Yes	No	Yes	No	
Male	20(60.6)	13(39.4)	6(18.2)	27(81.8)	22(66.7)	11(33.3)	4(12.1)	29(87.9)	6(18.2)	27(81.8)	33(100)
Female	20(57.1)	15(42.9)	6(17.1)	29(82.9)	3(8.6)	32(91.4)	1(2.9)	34(97.1)	3(8.6)	32(91.4)	35(100)
Total	40(58.8)	28(41.2)	12(17.6)	56(82.4)	25(36.8)	43(63.2)	5(7.4)	63(92.6)	9(13.2)	59(86.8)	68(100)
P - value	0.809		0.911		**0.000**		0.191		0.299		

Analysis with Chi-Square test of independence

More than half (55.9%, n = 38) of the respondents got embarrassed to a sexually suggestive advertisement and pornography shown by a friend (57.4%, n = 39) (Table 6). However 38.2% (n = 26) smiled at a kissing scene in a movie. Gender was found to have a significant (p = 0.038) relationship with respect to reaction to a kissing scene in a movie. More male respondents smiled to a kissing scene in a movie while more females got embarrassed. The majority (73.5%, n = 50) of the respondents indicated that none of their friends masturbate although a fifth (20.6%, n = 14) indicated that a few of their friends do. More males had a few of their friends that masturbate than females although the difference was not statistically significant. About 38.2% (n = 28) of the respondents knew of their friends who indulged in sex.

**Table 6:** Reactions to sexual exposure

Characteristic	Smile (%)	Embarrassment (%)	Desire to watch again (%)	Irresistible (%)	Total (%)	P- value
Reaction to a sexually suggestive advertisement						0.57
Male	4(12.1)	19(57.6)	6(18.2)	4(12.1)	33(100)	
Female	7(20)	19(54.3)	3(8.6)	6(17.1)	35(100)	
Total	11(16.2)	38(55.9)	9(13.2)	10(14.7)	68(100)	
	Reaction to a kissing scene in a movie					0.038
Male	18(54.5)	6(18.2)	5(15.2)	4(12.1)	33(100)	
Female	8(22.9)	15(42.9)	8(22.9)	4(11.4)	35(100)	
Total	26(38.2)	21(30.9)	13(19.1)	8(11.8)	68(100)	
	Reaction to a pornography shown by a friend					0.128
Male	7(21.2)	16(48.5)	6(18.2)	4(12.1)	33(100)	
Female	1(2.9)	23(65.7)	6(17.1)	5(14.3)	35(100)	
Total	8(11.8)	39(57.4)	12(17.6)	9(13.2)	68(100)	

Analysis with Chi-Square test of independence

## Discussion

Adolescents require being instilled with positive sexual values and parents can play a big part in influencing their sexual behaviors. Indeed, parents are expected to be the source of accurate information about sexuality and sexual matters^14^. The findings of this study indicate that it is the peers who inform the youngsters on matters of sexuality. As they mature, they realize the importance of getting accurate education on sex. This is when they wish that their parents had talked to them. These findings correspond with research results found in Botswana where almost twice of the adolescents were informed about sexuality matters by peers as compared to parents^15^. It has been suggested that early guidance by parents prepares a child for relationships^16^. Parents should consider being at the forefront in conveying sexual information to adolescents at an early age. This will have a positive influence on their sexual relationships.

Instilling morality and ethics in young ones makes them good people. Moral education provides intellectual resources that enable one to make informed decisions on sexual behavior^17^. The findings of this research indicate that moral and ethical education plays a big part in preventing adolescents from irresponsible sexual behavior. Ethics anchored on strong foundation of responsibility enables adolescents to weigh their sexual behavior correctly.

Source of sex information may have an influence on sexual behavior. For instance, sex education in school as a source of information has been associated with lower reporting of sexual health risk behaviors^18^. This study has found that internet is an easily accessible method for searching information which is then shared among friends. The adolescent tendency of taking risk and showing supremacy seems to come into play. Internet is a source of information to adolescents as they are more likely to go online. However, a number of issues can be raised on use of internet as a source of sex information. The sex information available online can be misleading^19^. It seems that there is peer pressure when it comes to sexual activities. However, the clear relationship between moral training and abstinence has not been captured but it may be of importance. The natural reluctance of the feminine gender is clearly captured in their non-indulgence in type of sexual behavior and reaction to sexual exposure. Surprisingly, all the respondents were not concerned with unwanted pregnancies, incurable diseases, loss of focus and losing virginity. Some of the reasons could be the easy access to birth control and other preventive measures.

The study shows that a large number of adolescents get exposure to sexual behavior at a very young age. Although a high proportion like love, the same majority does not seem to like marriage. This is not explained clearly from this survey. Pornography, masturbation and homosexuality are disliked by a vast majority indicating a conservative upbringing. The survey does not explain the fear of marriage among the females. It seems that the so called modern lifestyle represented by the public advertisements is not in line with prevalent cultural norms. The marketing tools employed for many products caused embarrassment to the youth especially the sexually suggestive tones. Similar observation can be drawn from the public entertainment scene. It is very clear from the survey that peers have a lot of influence inadvertently misguiding the youth. It is like the blind guiding the blind. The responsibility of the parents and the church cannot be over emphasized. The church should become aggressive in imparting moral values and the parents should not shy away from discussing sexually sensitive matters.

## Limitations

The sample size for the study was relatively small. Although it represents the heterogeneous population of Kenya, a bigger sample would do more justice. A follow up with more indirect questions to reaffirm the respondents' views could add to authenticity.

## Conclusion and study implications

The socio-economic scene is changing fast in Kenya. The old order does not hold any more. The parents and teachers have not yet fully comprehended what the adolescents are going through. There is a need for accurate sexual knowledge but there is confusion about who should provide it. Of course this is a common responsibility of parents, teachers and the church. However there is a need for the same study to be conducted on a national scale.

The findings throw a new light on the way modern Kenyan youth understand and attempt to solve issues. It tells the stake-holders to take the issue of adolescent sexuality seriously. For the parents, teachers and clergy it implies that all is not well. The findings from this study clearly indicate that in order to positively influence these adolescents perspective with regards to sexuality and sexual behaviour, it will take effort from clergy, parents and teachers to provide information required to influence attitudes and behaviour of these young people. This will be done through provision of appropriate sexuality education.
